# Supplementary material for: Low-frequency ionic-electronic coupling for energy-efficient noise-resilient wireless bioelectronics
Source: Nat Commun. 2026 Mar 11;17:3800. doi: 10.1038/s41467-026-70331-4 (PMC13111683; doi:10.1038/s41467-026-70331-4)
Supplement: Supplementary file 1 — Supplementary Information [file 41467_2026_70331_MOESM1_ESM.pdf]

Supplementary Information

Low-frequency ionic-electronic coupling for energy-efficient noise-resilient wireless bioelectronics

Ji Hong Kim[1]†, Haerim Kim[2]†, Jaewon Rhee[2], Joo Sung Kim[1‡], Hanbin Choi[1], Won Hyuk Choi[1], Yoseph Park[1], Jong Hwi Kim[1], So Young Kim[1], Seungyoung Ahn[2*], and Do Hwan Kim[1,3,4*]

[1]*Department of Chemical Engineering, Hanyang University, Seoul 04763, Republic of Korea.*
[2]*Cho Chun Shik Graduate School of Mobility, Korea Advanced Institute of Science and Technology, Daejeon 34051, Republic of Korea.*
[3]*Institute of Nano Science and Technology, Hanyang University, Seoul 04763, Republic of Korea.*
[4]*Clean-Energy Research Institute, Hanyang University, Seoul 04763, Republic of Korea.*

†These authors contributed equally to this work.
‡Current address: Thin-Film Device Laboratory, RIKEN, 2-1 Hirosawa, Wako, Saitama 351-0198, Japan.

*Corresponding authors' email: sahn@kaist.ac.kr (S.A.), dhkim76@hanyang.ac.kr (D.H.K.)

This PDF file includes:

Supplementary Figures 1 to 23

Supplementary Table 1 to 4

Supplementary Figures

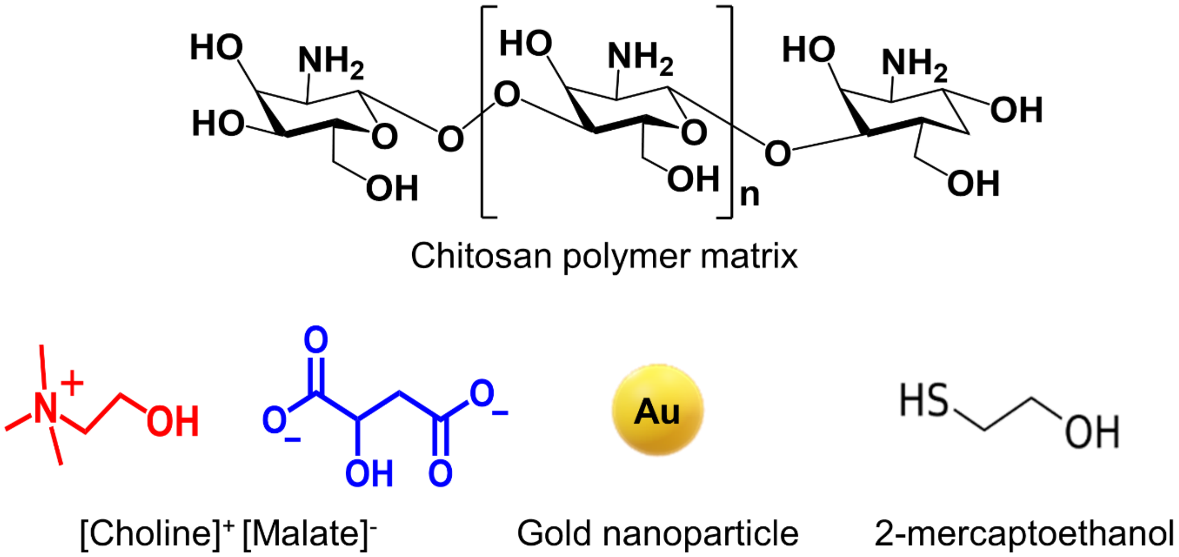

Supplementary Fig. 1 | Molecular structures of the wireless low-frequency electrochemical sensor

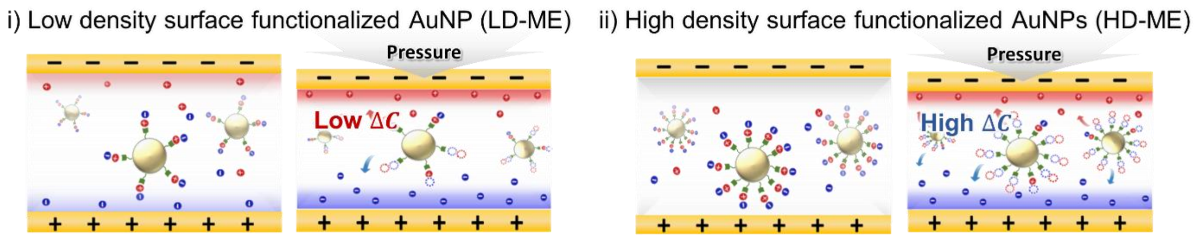

Supplementary Fig. 2 | Comparison of capacitance change induced by pressure in low (left) and high (right) functionalization surfaces of gold nanoparticles. The surface with high functionalization density shows a significantly higher capacitance change (ΔC) compared to the low functionalization surface, resulting in enhanced sensitivity.

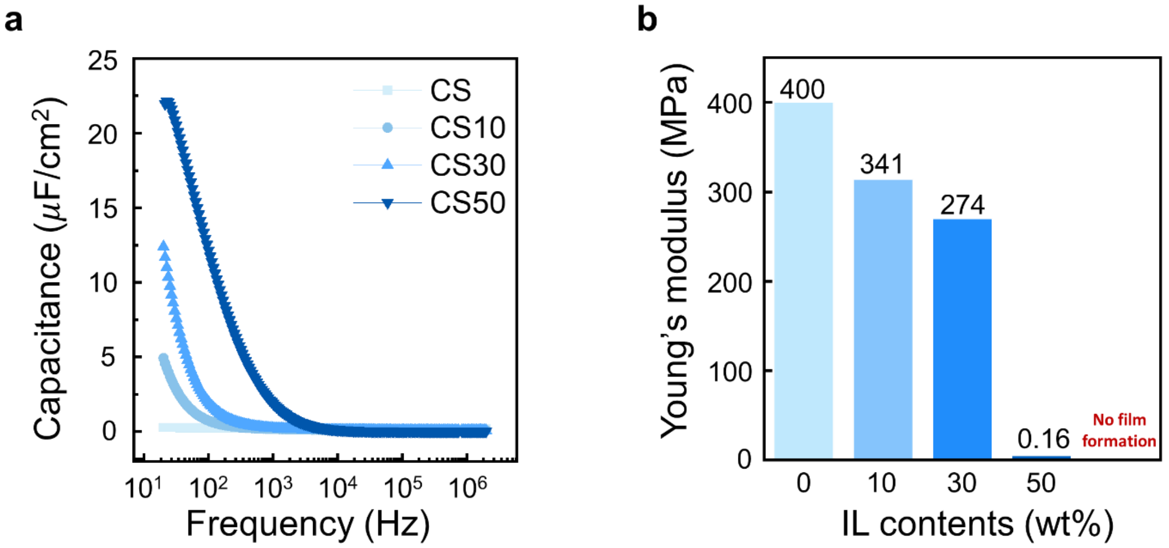

Supplementary Fig. 3 | Electrical and mechanical properties of chitosan-based ion gel **a,** Frequency-dependent capacitance of chitosan pristine ion gel with various ionic liquid content (0-50wt%). **b,** Young's modulus of chitosan pristine ion gel with various ionic liquid content (0-50wt%).

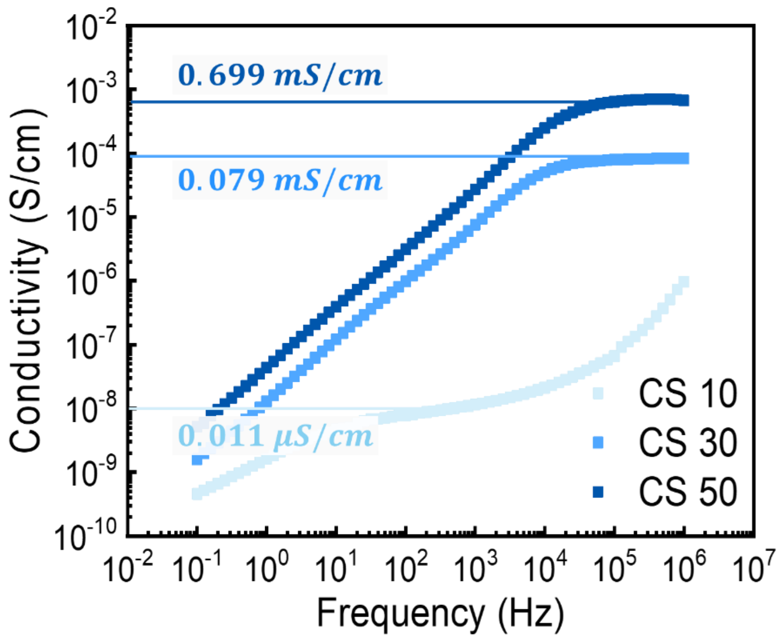

Supplementary Fig. 4 | Ionic conductivity from bode plots for pristine ion gel with various ion contents. As the ionic liquid content increases from 10 wt% to 50 wt%, the ionic conductivity increases dramatically.

a

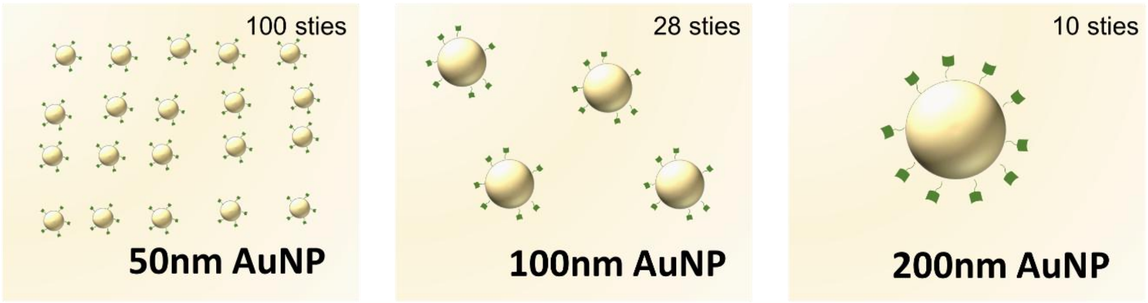

b

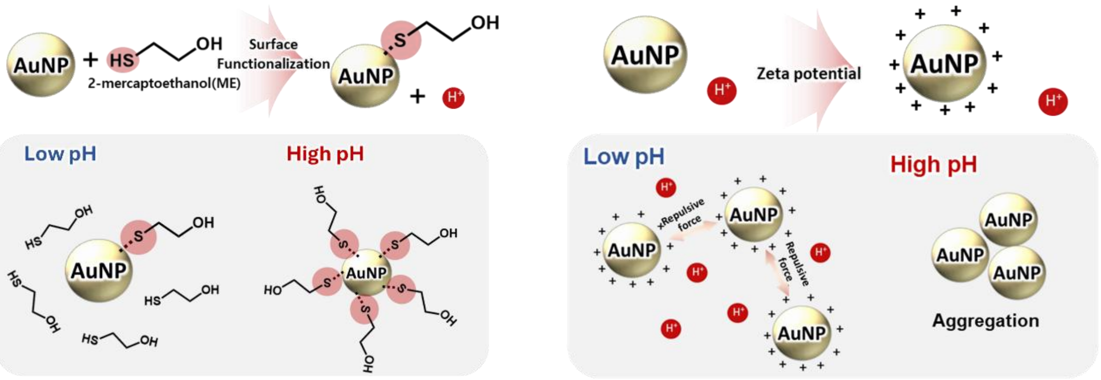

Supplementary Fig. 5 | Differences of surface functionalized density by (a) size of gold nanoparticle, and (b) synthetic pH condition.

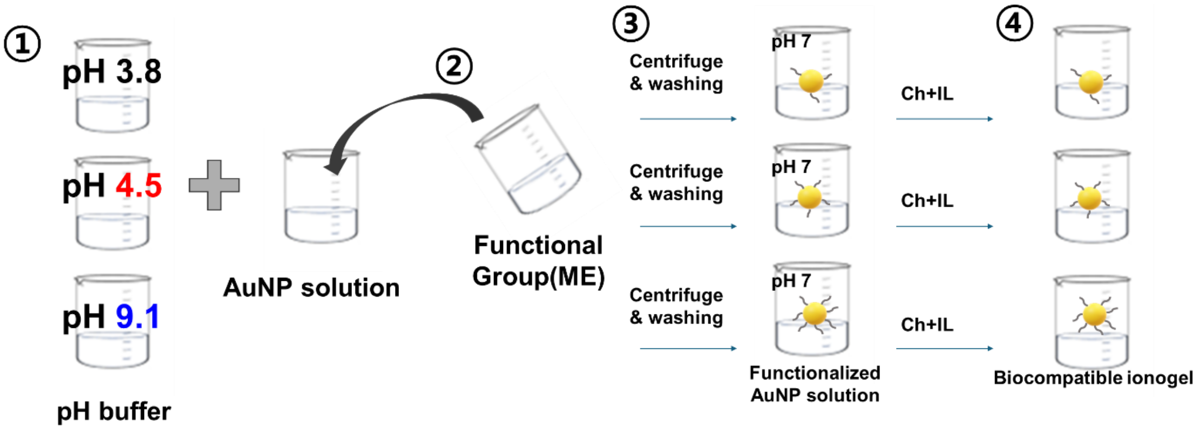

Supplementary Fig. 6 | Synthesis of functionalized gold nanoparticles. Schematics illustrates the synthesis process of functionalized gold nanoparticles.

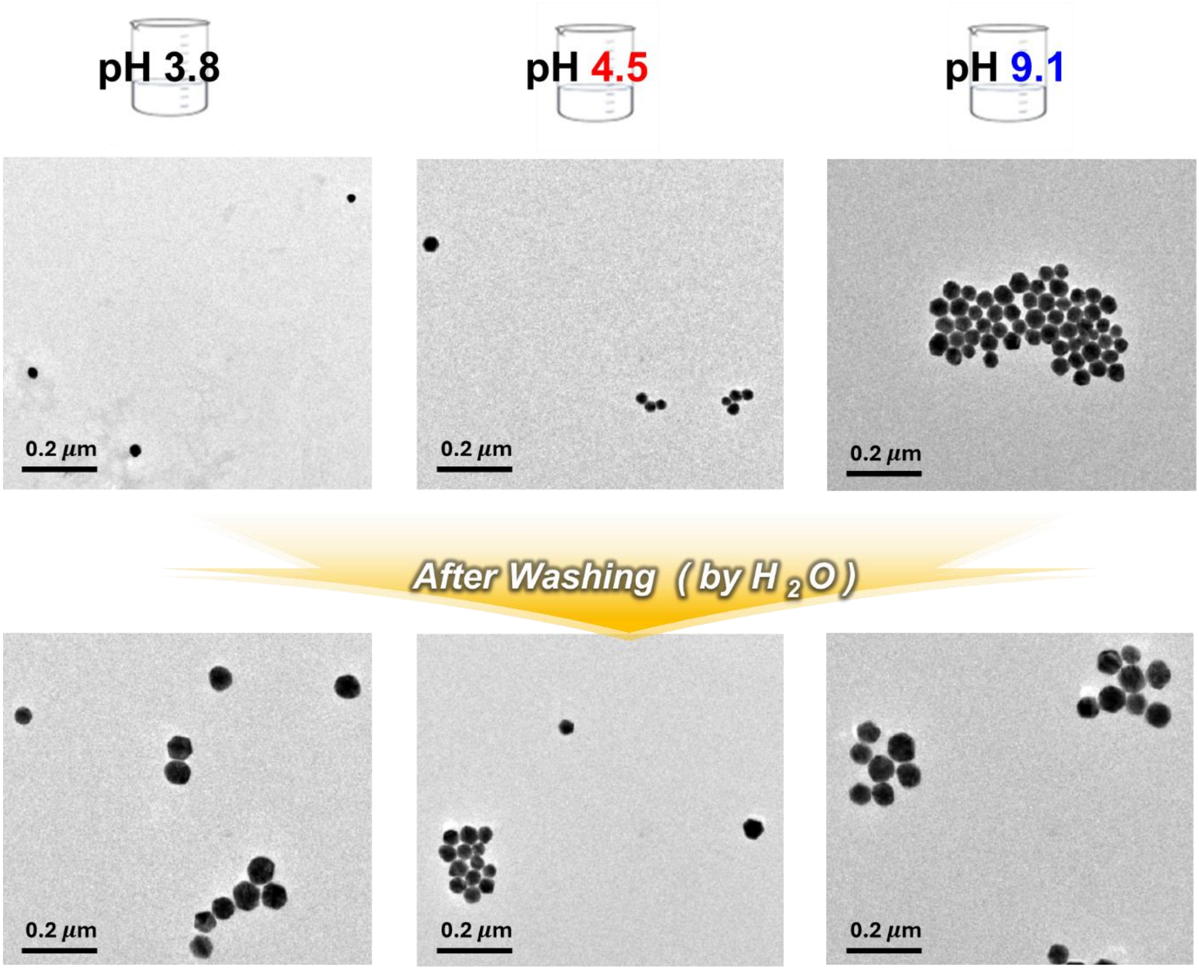

Supplementary Fig. 7 | TEM images of gold nanoparticles under different conditions displaying the aggregations of gold nanoparticles by varying pH conditions.

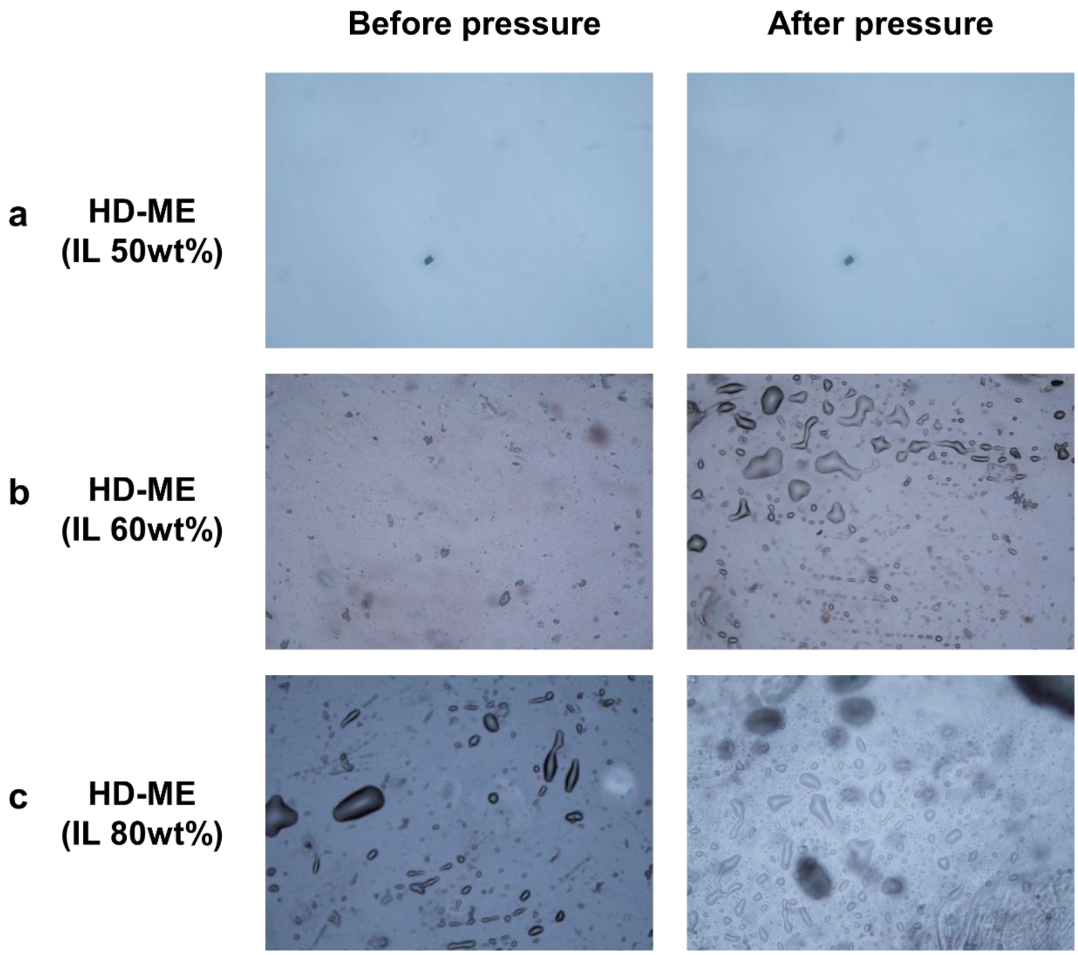

Supplementary Fig. 8 | Optical microscopy images of HD-ME under different ionic liquid (IL) contents: **a,** 50 wt% IL, **b,** 60 wt% IL, and **c,** 80 wt% IL, before and after pressure application.

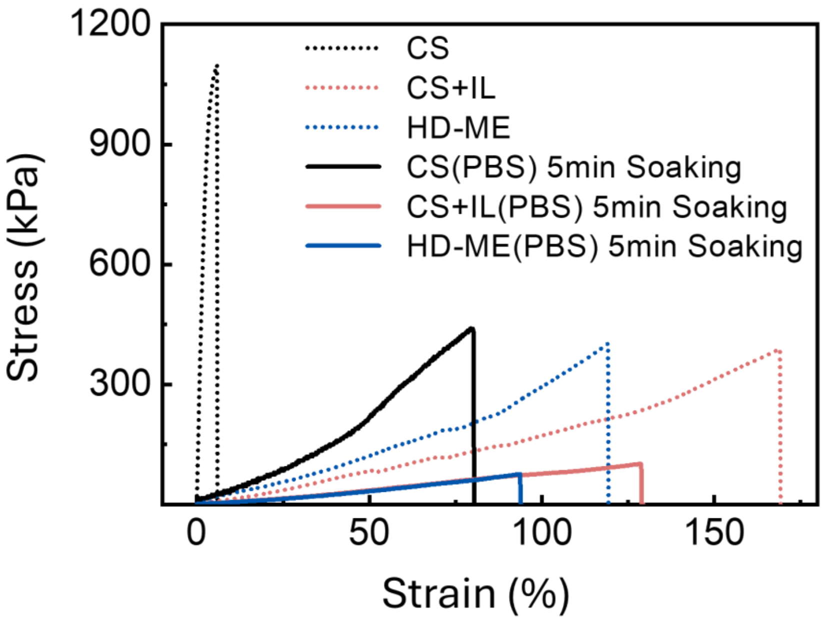

Supplementary Fig. 9 | Stress–strain curves of pristine chitosan(CS) gel, chitosan with ionic liquid(CS+IL) ion gel, and high-density surface functionalized gold nanoparticle(HD-ME) ion gel comparing before and after soaking in PBS buffer.

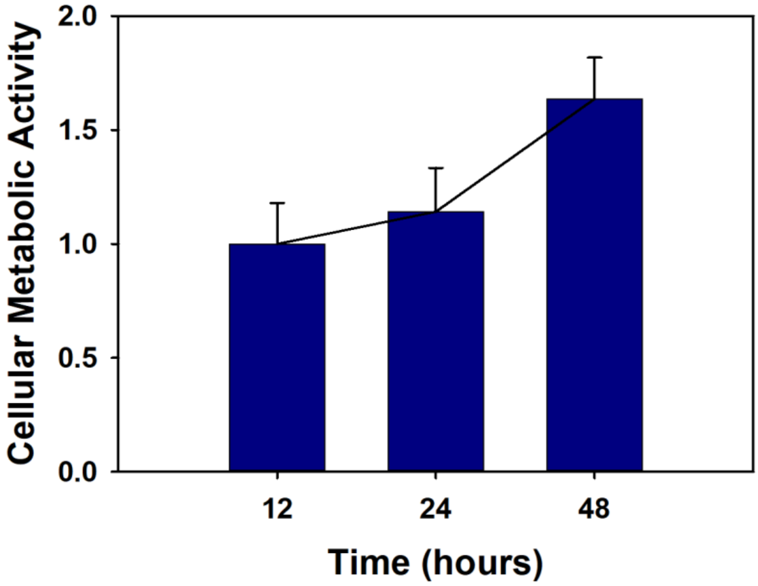

Supplementary Fig. 10 | Cellular metabolic activity of HD-ME ion gel over time (12-48 hours).

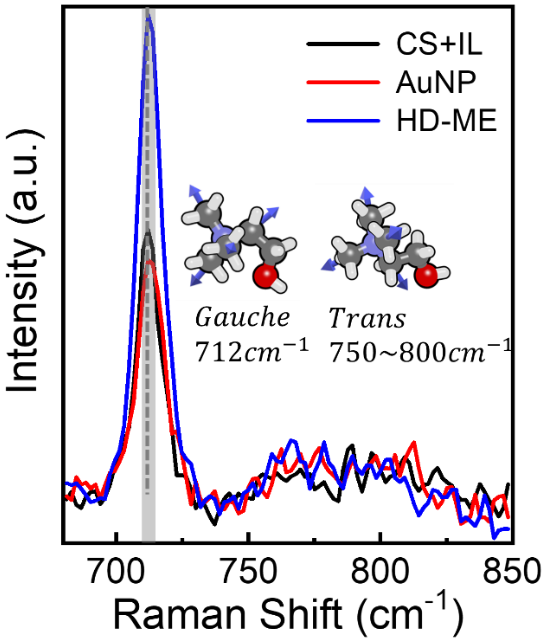

Supplementary Fig. 11 | The Raman spectra in the spectral range 680–850 cm^{-1} corresponding to choline cation conformation in various ion gel.

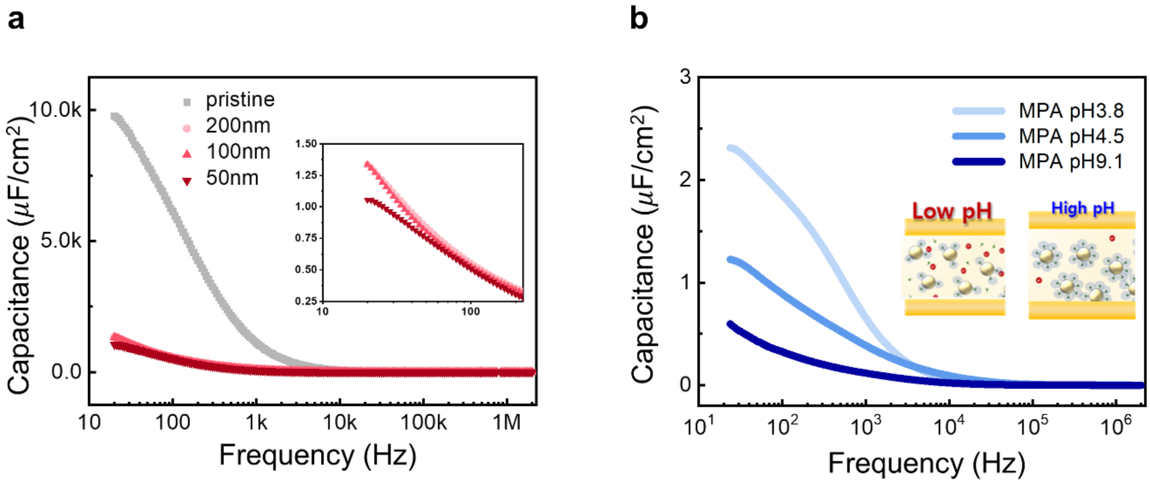

Supplementary Fig. 12 | Comparison of capacitance change as a function of frequency. The capacitance change was analyzed by different functionalized condition **a,** gold nanoparticle size (50-200nm) and **b,** synthesizing pH condition (pH 3.8-pH9.1) The capacitance change to frequency decrease with higher surface functionalization density.

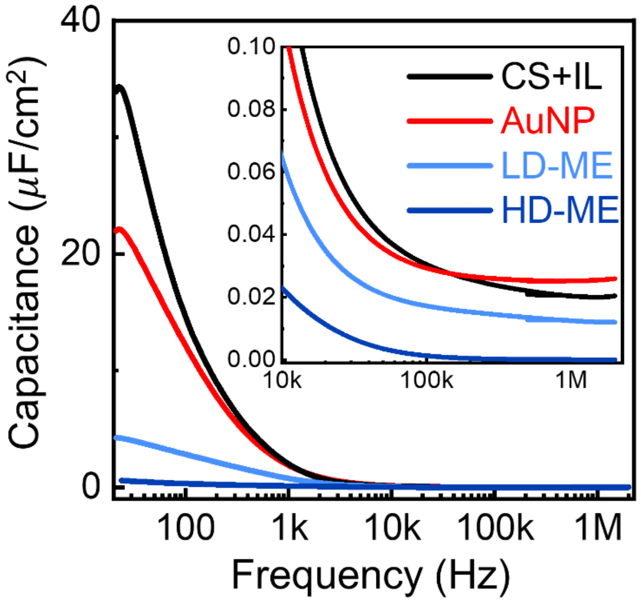

Supplementary Fig. 13 | Frequency vs. capacitance plots of various material-based ion gels. The strong ion-trapping effect of gold nanoparticles results in lower capacitance across various frequency ranges (20-2 MHz).

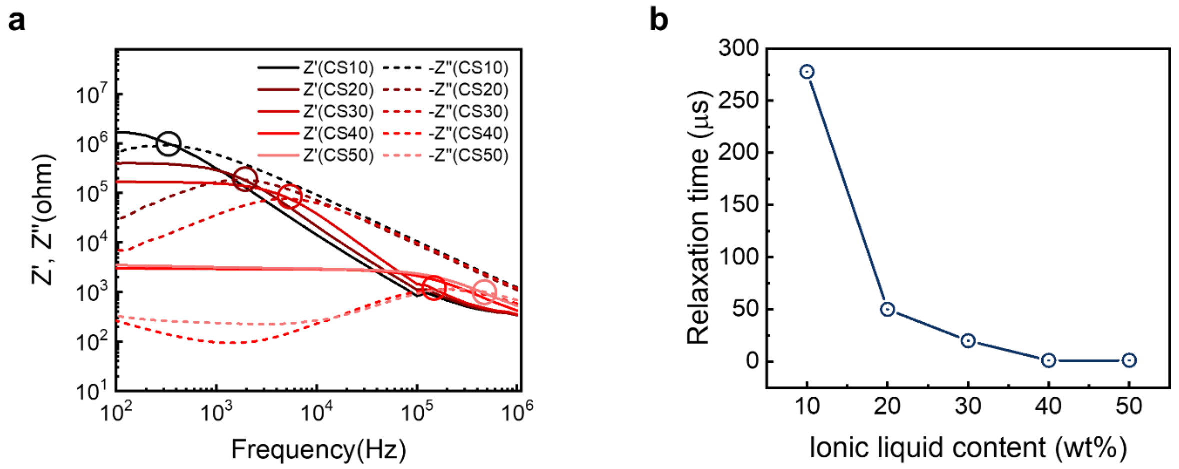

Supplementary Fig. 14 | Ion dynamics of chitosan-based ion gel. a, Frequency-dependent impedance spectra of the pristine ion gel, showing real (solid line) and imaginary (dashed line) components at varying ionic concentrations. **b,** Relaxation time values corresponding to different ionic liquid contents, derived from the impedance spectra.

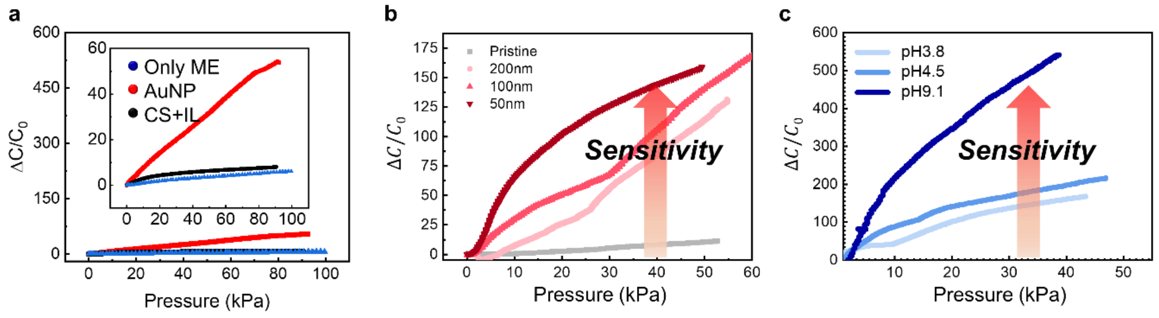

Supplementary Fig. 15 | Comparison of capacitance change as a function of applied pressure. The capacitance change was analyzed by different functionalized condition **a,** Different particle condition, **b,** gold nanoparticle size (50-200nm), and **c,** synthesizing pH condition (pH 3.8-pH 9.1). The sensitivity of capacitance changes to pressure increases with higher surface functionalization density.

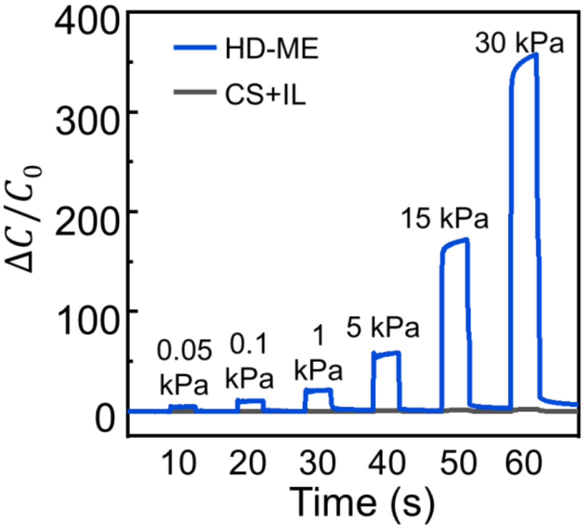

Supplementary Fig. 16 | Performance of pressure sensor based ion gels. Plots of relative change in capacitance as a function of applied pressures (0.05kPa, 0.1kPa, 1kPa, 5kPa, 15kPa, and 30kPa) comparison between HD-ME and CS+IL iongel.

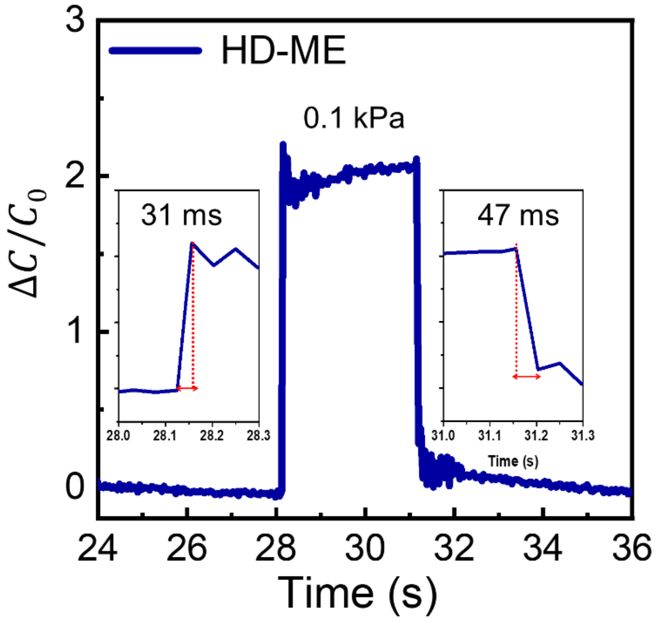

Supplementary Fig. 17 | Dynamic response of HD-ME ion gel based ionic capacitive pressure sensor. Transient response of HD-ME ion gel based ionic capacitive pressure sensor under 0.1 kPa. The inset shows a magnified curve representing a response time of 31 ms and a reset time of 47 ms, applied bias at 1V at 100 kHz.

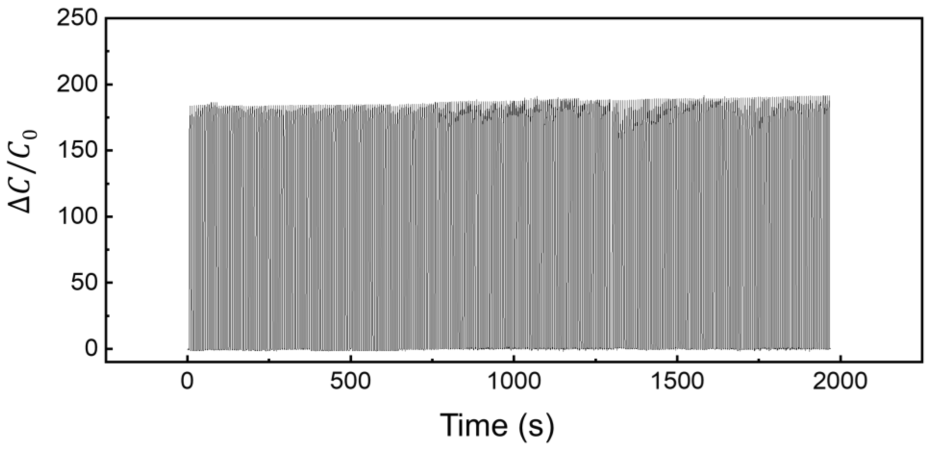

Supplementary Fig. 18 | Reliability of HD-ME ion gel based ionic capacitive pressure sensor. Mechanical durability test results of the HD-ME ion gel based sensor (700 cycles) applied bias at 1V at 100 kHz.

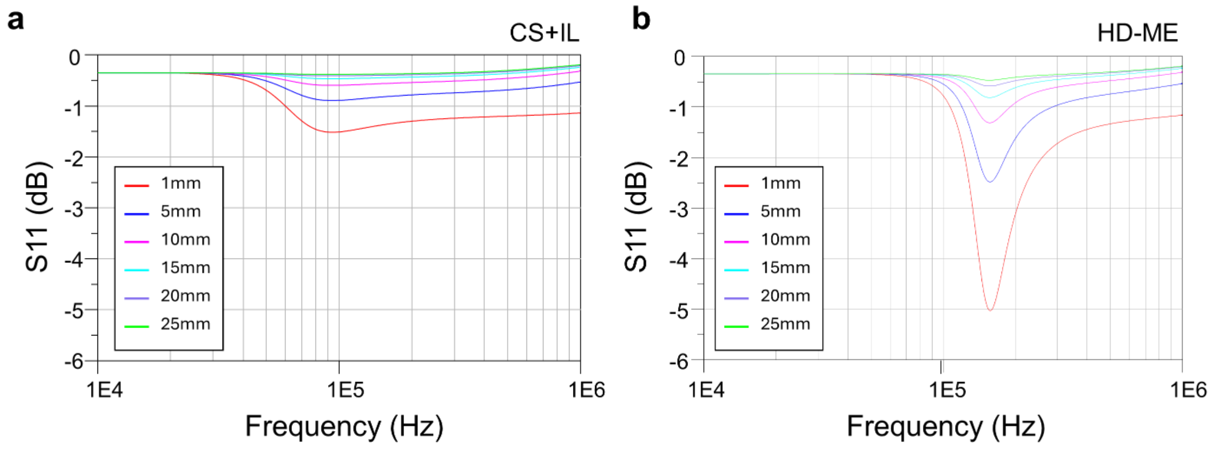

Supplementary Fig. 19 | Simulation results of sensitivity according to the air gap between the sensor coil and the reader coil (**a**) CS+IL ion gel, (**b**) HE-ME ion gel.

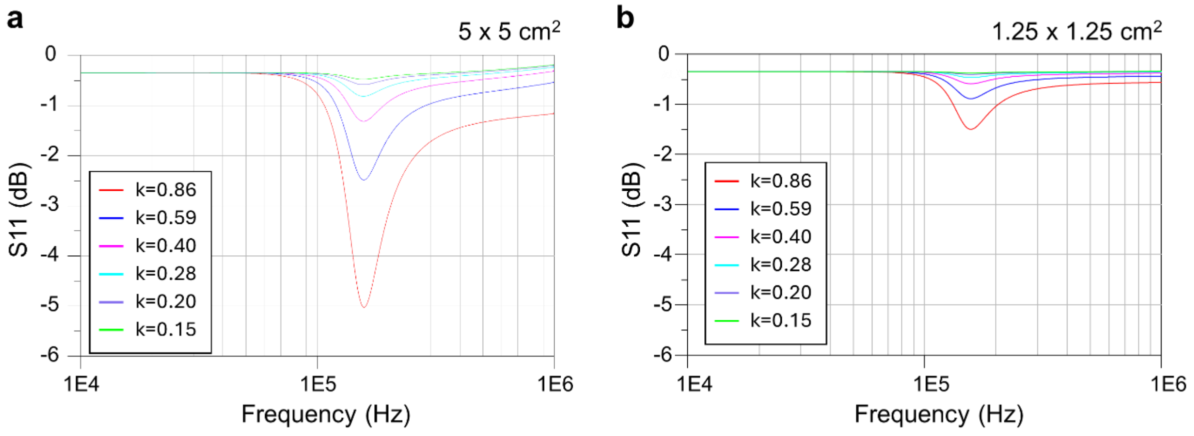

Supplementary Fig. 20 | Comparison of measurement sensitivity based on antenna size (**a**) 5 x 5 cm^2 and (**b**) 1.25 x 1.25 cm^2.

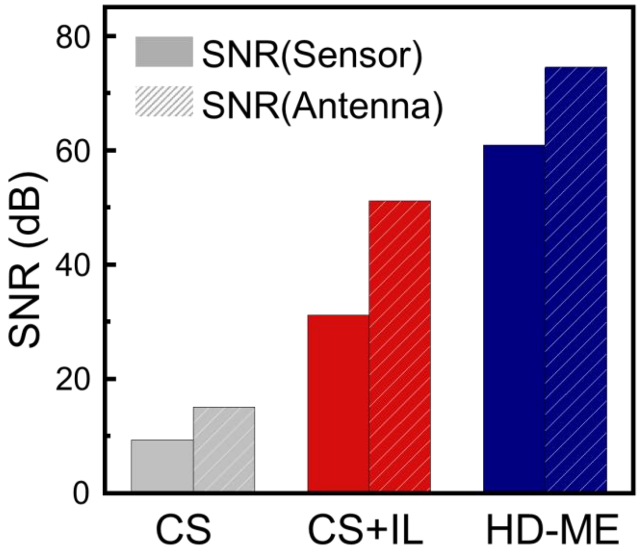

Supplementary Fig. 21 | Signal to noise ration of WiLECS platform with various materials.

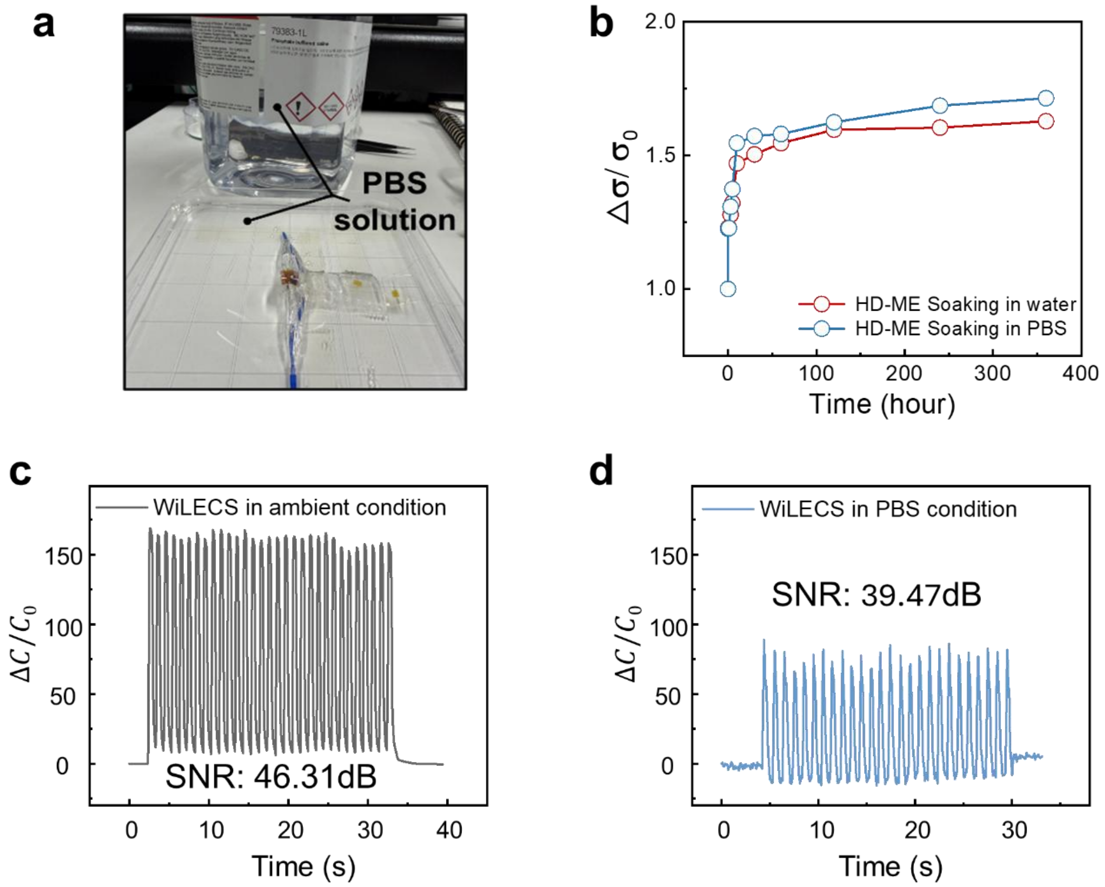

Supplementary Fig.22 | Signal-to-noise ratio (SNR) of WiLECS under physiologically relevant conditions. a, Photograph of the WiLECS in PBS solution **b,** Multi-days relative conductivity change plot for ion leaching test **c,** Relative capacitance change plots of WiLECS in ambient condition **d,** Relative capacitance change plots of WiLECS in PBS condition after 15days.

a b c

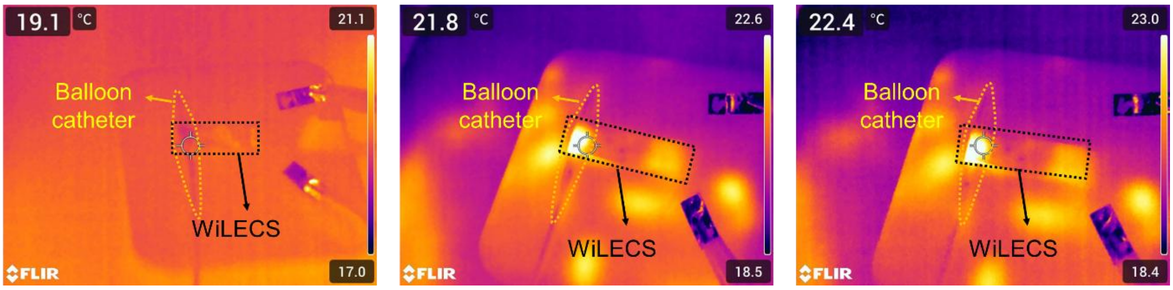

Supplementary Fig.23 | Thermal characterization of the WiLECS during wireless operation. Infrared thermal images of the WiLECS device recorded before **a,** wireless operation, **b,** during steady-state operation, and **c,** after 6 hours operation, measured using a thermal imaging camera under the same operating conditions as the wireless sensing experiments.

Supplementary Tables

Supplementary Table. 1 | Quantitative comparison of reported wireless pressure sensors, summarizing their sensitivity and operating frequency ranges.

Ref	Materials	Sensitivity	Frequency range
7	Patterned PGS	$0.000196 mmHg^{-1}$	110-90MHz
8	Patterned PDMS	$0.000439 mmHg^{-1}$	5-30MHz
23	Patterned ZnO/PLCL capacitive	$0.002705 mmHg^{-1}$	250-300MHz
24	Patterned PDMS capacitive	$0.00146 mmHg^{-1}$	250-300MHz
25	Eco flex	$0.00122 mmHg^{-1}$	3-5GHz
26	PI air cavity	$0.000712 mmHg^{-1}$	10-20MHz
27	Eco flex	$0.0008254 mmHg^{-1}$	200-350MHz
28	Parallel-plate(air cavity)	$0.0006316 mmHg^{-1}$	100-200MHz
29	Fabric spacer (air cavity)	$0.000575757 mmHg^{-1}$	30-45MHz
30	Parallel-plate(air cavity)	$0.0003017 mmHg^{-1}$	300-400MHz

Supplementary Table. 2 | Simulation results of sensitivity according to the air gap between the sensor coil and the reader coil.

CS+IL		HD-ME	
Gap [mm]	S_{11} [dB]	Gap [mm]	S_{11} [dB]
1	1.52	1	5.03
5	0.90	5	2.48
10	0.60	10	1.32
15	0.47	15	0.82
20	0.41	20	0.58
25	0.38	25	0.48

Supplementary Table. 3 | Comparison of measurement sensitivity based on antenna size.

5 x 5 cm^2			1.25 x 1.25 cm^2		
Gap [mm]	Coupling coefficient	S_{11} [dB]	Gap [mm]	Coupling coefficient	S_{11} [dB]
1	0.86	5.03	1	0.86	1.50
5	0.59	2.48	5	0.59	0.89
10	0.40	1.32	10	0.40	0.60
15	0.28	0.82	15	0.28	0.47
20	0.20	0.58	20	0.20	0.41
25	0.15	0.48	25	0.15	0.38

Supplementary Table. 4 | Quantitative Comparison of Initial Capacitance, Pressure-Induced Capacitance Modulation, Resonance Shift, and SNR Across Different Gel Capacitor Material (ΔP = 0–50 kPa).

	CS gel	CS+IL ion gel	HD-ME ion gel
Initial C (μF/cm²)	3.75×10^{-2}	34.203	0.59663
$\Delta C/C_0$	0.20994	6.3019	392.82189
Δf (kHz)	-7.313	-33.29	-192.6
SNR (dB)	9.3023	31.121	60.94
Sensitivity ($mmHg^{-1}$)	5.60×10^{-4}	0.0168	1.047
