# Supplementary material for: Low-frequency ionic-electronic coupling for energy-efficient noise-resilient wireless bioelectronics
Source: Nat Commun. 2026 Mar 11;17:3800. doi: 10.1038/s41467-026-70331-4 (PMC13111683; doi:10.1038/s41467-026-70331-4)
Supplement: Supplementary file 2 — Description of Additional Supplementary Information [file 41467_2026_70331_MOESM2_ESM.pdf]

Description of Additional Supplementary Files

File Name: Supplementary Movie 1
Description: Wireless transmission of WiLECS integrated with a balloon catheter. This movie presents the overall view of the wireless operation of WiLECS.

File Name: Supplementary Movie 2
Description: Wireless transmission of WiLECS through Teflon spacer. This movie presents the overall view of the wireless operation of WiLECS through Teflon spacer.

File Name: Supplementary Movie 3
Description: Wireless transmission of WiLECS through porcine skin. This movie presents the overall view of the wireless operation of WiLECS through porcine skin.
